# Diversity of *Wickerhamomyces* (Wickerhamomycetaceae, Saccharomycetales) in China with the description of four new species

**DOI:** 10.3389/fmicb.2024.1338231

**Published:** 2024-02-08

**Authors:** Chun-Yue Chai, Tao Ke, Qiu-Hong Niu, Feng-Li Hui

**Affiliations:** ^1^School of Life Science and Agricultural Engineering, Nanyang Normal University, Nanyang, China; ^2^Research Center of Henan Provincial Agricultural Biomass Resource Engineering and Technology, Nanyang Normal University, Nanyang, China

**Keywords:** Ascomycetes, morphology, phylogenetic analyses, taxonomy, plants, tropical and subtropical regions

## Abstract

*Wickerhamomyces* is a well-known genus of the family Wickerhamomycetaceae in the class Ascomycetes. These fungi can survive in a variety of substrates and environments and perform many valuable roles in both industrial processes and the natural ecosystems. During our investigation of yeast diversity associated with plant materials, 53 *Wickerhamomyces* isolates were obtained from rotting wood and plant leaves collected in Fujian, Guizhou, Henan, and Yunnan Provinces of China. Isolates were identified as 14 *Wickerhamomyces* species, including 1 species known previously to occur in China (*W. anomalus*), 9 new record species in China (*W. arborarius*, *W. ciferrii*, *W. edaphicus*, *W. lynferdii*, *W. pijperi*, *W. subpelliculosa*, *W. xylosica*, *W. strasburgensis*, and *W. sydowiorum*), and 4 novel species (*W. guiyangensis* sp. nov., *W. paramyanmarensis* sp. nov., *W. quanzhouensis* sp. nov., and *W. phyllophilus* sp. nov.). This study presents a detailed account of these new species, illustrating their morphology and analyzing their phylogenetic relationships with other *Wickerhamomyces* species. Our study is the first comprehensive study on *Wickerhamomyces* species associated with plant materials from tropical and subtropical China. The results of this study update our understanding of the phylogenetic relationships, systematics, and ecology of *Wickerhamomyces*.

## 1 Introduction

The genus *Wickerhamomyces* was first established by Kurtzman et al. (2008) with the type species *Wickerhamomyces canadensis*, which formerly belonged to the genus *Pichia* (Kurtzman, 2011a). Based on the phylogenetic analysis of concatenated sequences of LSU, SSU, and *EF-1α*, this genus was placed in the family *Wickerhamomycetaceae* of the order Saccharomycetales (Kurtzman et al., 2008), which was further confirmed by using a genome-scale phylogeny (Groenewald et al., 2023). With the progress of yeast isolation technology and molecular biology, a number of *Wickerhamomyces* species have been discovered in different regions of the world. Currently, *Wickerhamomyces* consists of 38 defined species, including the most recent additions of *W. lannaensis*, *W. nanensis*, and *W. sinyiensis* (Nundaeng et al., 2021; Chang et al., 2022). Among the 38 valid species, 12 are asexual morphs and 26 have known for their ascosporic states (Kurtzman, 2011b; Shimizu et al., 2020; Nundaeng et al., 2021; Chang et al., 2022). In addition, 10 anamorphic *Candida* species, including *C. mycetangii*, *C. namnaoensis*, *C. nitrativorans*, *C. odintsovae*, *C. peoriensis*, *C. ponderosae*, *C. quercuum*, *C. silvicultrix*, *C. solani*, and *C. ulmi*, belong to the *Wickerhamomyces* clade based on phylogenetic analysis (Kurtzman, 2011b; Lachance et al., 2011; Shimizu et al., 2020), which will be transferred to the genus *Wickerhamomyces* as new combinations in the future. Phenotypically, the asexual morphs of *Wickerhamomyces* reproduced by multilateral budding on a narrow base, and some species produce pseudohyphae and/or true hyphae (Shimizu et al., 2020; Nundaeng et al., 2021; Chang et al., 2022). The sexual morphs of *Wickerhamomyces* are characterized by the production of hat-shaped or spherical ascospores with equatorial ledges (Kurtzman et al., 2008; Kurtzman, 2011b). Members of the genus *Wickerhamomyces* ferment glucose and possess Q-7 as a predominant ubiquinone. They can utilize various carbon sources but not methanol or hexadecane (Kurtzman et al., 2008; Kurtzman, 2011b). Due to the similarity in phenotypic characteristics within the genus, a combination of phenotypical characteristics and phylogenetic analysis has been adopted as the standard method for concretely identifying *Wickerhamomyces* species (Kurtzman et al., 2008; Nundaeng et al., 2021).

Members of the genus *Wickerhamomyces* have been studied for a variety of applications, and most previous studies have focused on the most widely distributed species *W. anomalus* (Nundaeng et al., 2021). The strains of this species offer a diversity of desirable characteristics; BS91 and DMKU-RP04 demonstrated resistance to plant pathogenic fungi (Czarnecka et al., 2019; Limtong et al., 2020); SDBR-CMU-S1-06 and Wa-32 produced the plant growth promoter indole-3-acitic acid (IAA) (Kumla et al., 2018); CBS261, HN006, and HN010 produced ethylacetate, which is used to enhance the aroma and quality of Chinese liquor (Ravasio et al., 2018; Yan et al., 2019; Li et al., 2021). Notably, most strains of *W. anomalus* are known to produce killer toxins that possess antimicrobial and larvicidal activities (Walker, 2011; Nundaeng et al., 2021). In addition, several other species, including *W. bovis*, *W. silvicola*, *W. edaphicus*, *W. siamensis*, *W. rabaulensis*, *W. psychrolipolyticus*, and *W. chambardii*, can be produced mycocin, biosurfactants, saturated fatty acids, xylitol, cellulase, and lipases that could be applied in the bioremediation, biotechnology, food, and cosmetic industries (Shimizu et al., 2020; Nundaeng et al., 2021). On the other hand, some *Wickerhamomyces* species, such as *W. anomalus* and *W. lynferdii*, have been responsible for the spoilage of beer and bakery products (Lanciotti et al., 1998; Timke et al., 2005). *W. anomalus*, *W. myanmarensis*, and *W. onychis*, which have been reported to exist clinical specimens, appear to be opportunistic human pathogens (Kurtzman, 2011b; Arastehfar et al., 2019).

Species of *Wickerhamomyces* are widely distributed in tropical, subtropical, and temperate regions (Kurtzman, 2011b; Nundaeng et al., 2021). Presently, 20 *Wickerhamomyces* species have been reported in Asia, with 10 species (*W. anomalus*, *W. ciferrii*, *W. edaphicus*, *W. lannaensis*, *W. nanensis*, *W. rabaulensis*, *W. siamensis*, *W. sydowiorum*, *W. tratensis*, and *W. xylosicus*) found in Thailand (Limtong et al., 2012; Nakase et al., 2012; Kaewwichian et al., 2013), two (*W. psychrolipolyticus* and *W. scolytoplatypi*) in Japan (Ninomiya et al., 2013; Shimizu et al., 2020), two (*W. silvicola* and *W. ochangensis*) in South Korea (Shin et al., 2011), one (*W. xylosivorus*) in Indonesia (Kobayashi et al., 2017), and four (*W. anomalus*, *W. kurtzmanii*, *W. menglaensis*, and *W. mori*) in China (Hui et al., 2013; You et al., 2016; Chai et al., 2019; Zhou et al., 2019).

The presence of *Wickerhamomyces* in China has not been extensively documented. Current members of the genus have been reported only in Sichuan Province (*W. anomalus*) (You et al., 2016), the Inner Mongolia Autonomous Region (*W. kurtzmanii*) (Zhou et al., 2019), Yunnan Province (*W. menglaensis*) (Chai et al., 2019), and Henan Province (*W. mori*) (Hui et al., 2013). China is located in the northern hemisphere, providing a temperate environment suitable for hosting *Wickerhamomyces* species. To investigate the diversity of *Wickerhamomyces* in China, we isolated yeast strains from plant materials collected in Fujian, Guizhou, Henan, and Yunnan Provinces using a culture-based approach. A total of 14 species, including 4 new species, were identified from the isolated yeast strains. This study aims to characterize the diversity of *Wickerhamomyces* species inhabiting these substrates and describe four new species based on morphological characteristics and phylogenetic analyses of ITS and LSU.

## 2 Materials and methods

### 2.1 Sampling and yeast isolation

Thirty-six strains used in this study were isolated from rotting wood by an enrichment method described by Shi et al. (2020). An additional 17 strains were isolated from plant leaves by the ballistospore-fall method as previously described (Nakase and Takashima, 1993; Hu et al., 2022). Vaseline was employed to adhere semi-withered leaf samples inside the lids of Petri plates containing yeast malt (YM) agar (0.3% yeast extract, 0.3% malt extract, 0.5% peptone, 1% glucose, and 2% agar). The plates were then incubated at 25°C for 7 days until colonies gradually formed. Different yeast morphotypes were picked and purified by streaking onto fresh YM agar. All purified yeast strains were then suspended in YM broth supplemented with 20% (v/v) glycerol and stored at −80°C. All isolates used in this study and their origins are presented in Table 1.

**Table 1:** Yeast strains and isolation sources investigated in this study.

Species	Strains	Sources	Location
*Wickerhamomyces anomalus*	NYNU 16830	Rotting wood	Neixiang, Henan, China
	NYNU 17762	Rotting wood	Chuxong, Yunnan, China
	NYNU 17785	Rotting wood	Chuxong, Yunnan, China
	NYNU 17881	Rotting wood	Nanzhang, Henan, China
	NYNU 177145	Rotting wood	Chuxong, Yunnan, China
	NYNU 177148	Rotting wood	Chuxong, Yunnan, China
	NYNU 177169	Rotting wood	Chuxong, Yunnan, China
	NYNU 177171	Rotting wood	Chuxong, Yunnan, China
	NYNU 177174	Rotting wood	Chuxong, Yunnan, China
	NYNU 177186	Rotting wood	Chuxong, Yunnan, China
*Wickerhamomyces arborarius*	NYNU 223273	Rotting wood	Quanzhou, Fujian, China
*Wickerhamomyces ciferrii*	NYNU 223281	Rotting wood	Quanzhou, Fujian, China
*Wickerhamomyces edaphicus*	NYNU 22329	Rotting wood	Quanzhou, Fujian, China
	NYNU 22331	Rotting wood	Quanzhou, Fujian, China
	NYNU 22333	Rotting wood	Quanzhou, Fujian, China
	NYNU 223131	Rotting wood	Quanzhou, Fujian, China
	NYNU 223132	Rotting wood	Quanzhou, Fujian, China
*Wickerhamomyces lynferdii*	NYNU 223170	Rotting wood	Quanzhou, Fujian, China
	NYNU 223193	Rotting wood	Quanzhou, Fujian, China
	NYNU 223197	Rotting wood	Quanzhou, Fujian, China
	NYNU 223280	Rotting wood	Quanzhou, Fujian, China
*Wickerhamomyces pijperi*	NYNU 17454	Rotting wood	Honghe, Yunnan, China
	NYNU 17874	Rotting wood	Nanzhao, Henan, China
	NYNU 17912	Rotting wood	Jinghong, Yunnan, China
	NYNU 20955	Rotting wood	Neixiang, Henan, China
	NYNU 20965	Rotting wood	Neixiang, Henan, China
	NYNU 178161	Rotting wood	Nanzhao, Henan, China
	NYNU 178245	Rotting wood	Zhaotong, Yunnan, China
*Wickerhamomyces* sp. 1	NYNU 22496	Rotting wood	Quanzhou, Fujian, China
	NYNU 224139	Rotting wood	Quanzhou, Fujian, China
	NYNU 224203	Rotting wood	Quanzhou, Fujian, China
*Wickerhamomyces* sp. 2	NYNU 2285	Phylloplane	Guiyang, Guizhou, China
	NYNU 2289	Phylloplane	Guiyang, Guizhou, China
*Wickerhamomyces* sp. 3	NYNU 2286	Phylloplane	Guiyang, Guizhou, China
	NYNU 2288	Phylloplane	Guiyang, Guizhou, China
	NYNU 22815	Phylloplane	Guiyang, Guizhou, China
	NYNU 22852	Phylloplane	Guiyang, Guizhou, China
*Wickerhamomyces* sp. 4	NYNU 22853	Phylloplane	Guiyang, Guizhou, China
*Wickerhamomyces strasburgensis*	NYNU 22837	Phylloplane	Guiyang, Guizhou, China
	NYNU 22851	Phylloplane	Guiyang, Guizhou, China
	NYNU 22991	Phylloplane	Guiyang, Guizhou, China
*Wickerhamomyces subpelliculosa*	NYNU 17431	Rotting wood	Honghe, Yunnan, China
*Wickerhamomyces sydowiorum*	NYNU 22810	Phylloplane	Guiyang, Guizhou, China
	NYNU 22825	Phylloplane	Guiyang, Guizhou, China
	NYNU 22826	Phylloplane	Guiyang, Guizhou, China
	NYNU 22827	Phylloplane	Guiyang, Guizhou, China
	NYNU 22850	Phylloplane	Guiyang, Guizhou, China
	NYNU 22856	Phylloplane	Guiyang, Guizhou, China
	NYNU 229153	Phylloplane	Guiyang, Guizhou, China
*Wickerhamomyces xylosica*	NYNU 22497	Rotting wood	Quanzhou, Fujian, China
	NYNU 224171	Rotting wood	Quanzhou, Fujian, China
	NYNU 224206	Rotting wood	Quanzhou, Fujian, China
	NYNU 224211	Rotting wood	Quanzhou, Fujian, China

### 2.2 Morphological and physiological characterization

The morphological and physiological characteristics of each yeast strain were examined according to the methods established by Kurtzman et al. (2011). Colony characteristics were observed on YM agar after 2 weeks of incubation at 25°C. Mycelium formation was investigated by cultivation on corn meal (CM) agar (2% cornmeal infusion and 2% agar) in slide culture at 25°C for 2 weeks. Sexual processes of all strains were studied individually and in strain pairs on YM agar, CM agar, 5% malt extract (ME) agar, and V8 agar at 20°C and 25°C for 2 months with weekly observation (Kurtzman, 2011b). Fermentation of glucose was carried out in liquid medium using Durham fermentation tubes. Carbon source and nitrogen source assimilation tests were conducted in liquid medium, and starved inoculum was used in nitrogen assimilation tests (Kurtzman et al., 2011). Cycloheximide resistance was performed in liquid medium, and urea hydrolysis was conducted on agar slants. Acid production and the diazonium blue B (DBB) reaction tests were performed in Petri dish with a solid medium. Growth potential at various temperatures (15°C, 20°C, 25°C, 30°C, 35°C, and 37°C) was determined by cultivation on YM agar. Cell morphology was examined through a Leica DM 2500 microscope (Leica Microsystems GmbH, Wetzlar, Germany) with a Leica DFC295 digital microscope color camera employing bright field, phase contrast, and differential interference contrast (DIC) optics. Novel taxonomic descriptions and proposed names were deposited in MycoBank (http://www.mycobank.org; 8 November 2023).

### 2.3 DNA extraction, PCR amplification, and sequencing

Genomic DNA was extracted from each yeast isolate using the Ezup Column Yeast Genomic DNA Purification Kit, according to the manufacturer’s protocol (Sangon Biotech, Shanghai, China). Sequenced data were generated from the internal transcribed spacer (ITS) region and the D1/D2 domain of the large subunit (LSU) rRNA gene using primer pairs ITS1/ITS4 (White et al., 1990) and NL1/NL4 (Kurtzman and Robnett, 1998). PCR amplification was performed in a 25 μL reaction volume containing 9.5 μL of ddH_2_O, 12.5 μL of 2 × Taq PCR Master Mix with blue dye (Sangon Biotech, Shanghai, China), 1 μL of DNA template, and 1 μL of each primer. PCR reactions were carried out according to the following conditions: initial denaturation step at 95°C for 2 min, followed by 35 cycles of 95°C for 30 s, 56°C for 30 s, 72°C for 40 s, and a final extension at 72°C for 10 min. PCR products were checked and purified in 1% agarose gels before being sequenced by Sangon Biotech (Shanghai) Co., Ltd. The identity and accuracy of each sequence were determined by GenBank sequences and assembled using BioEdit 7.1.3.0 (Hall, 1999). Newly obtained sequences were then submitted to GenBank (https://www.ncbi.nlm.nih.gov/genbank/; Table 2).

**Table 2:** Taxa, strains, sources, locations, and corresponding GenBank numbers of the taxa used in this study.

Species	Strains	Sources	Location	GenBank Accession No.	
				LSU D1/D2	ITS
*Candida mycetangii*	CBS 8675^T^	Mycetangia	USA	AF017241	NR_152475
*C. namnaoensis*	CBS 12175^T^	Insect frass	Thailand	AB607030	KY102224
*C. nitrativorans*	CBS 6152^T^	Fermented cocoa	Ivory coast	AJ508573	HM156506
*C. odintsovae*	CBS 6026^T^	Sap of *Betula verrucosa*	Russia	KY106610	NR_077084
*C. peoriensis*	CBS 8800^T^	Stump of *Ulmus* sp.	USA	NG_057166	NR_154960
*C. ponderosae*	CBS 8801^T^	Insect frass	USA	NG_057178	NR_154961
*C. quercuum*	CBS 6422^T^	Exudate of *Quercus serrata*	Japan	NG_057158	NR_154962
*C. silvicultrix*	CBS 6269^T^	Insect frass	South Africa	NG_057171	NR_165969
*C. solani*	CBS 1908^T^	Potato-starch mill	Netherlands	NG_055191	KY102402
*C. ulmi*	CBS 8670^T^	Insect frass	USA	NG_057160	—
*Wickerhamomyces alni*	CBS 6986^T^	Exudate of *Alnus rubra*	Canada	NG_057159	NR_154966
*W. anomalus*	CBS 5759^T^	—	USA	EF550341	EF550341
*W. arborarius*	CBS 12941^T^	Flower	Ecuador	NG_057179	NR_155000
*W. bisporus*	CBS 1890^T^	Insect frass	Austria	NG_057161	KY105897
*W. bovis*	CBS 2616^T^	Caecum of *Bos taurus*	Portugal	NG_057162	NR_154968
*W. canadensis*	NRRL Y-1888^T^	Beetle frass	Canada	NG_057163	—
*W. chambardii*	CBS 1900^T^	Chestnut	France	*NG_057177*	NR_154969
*W. chaumierensis*	CBS 8565^T^	Surface of flower	Guyana	NG_057180	HM156503
*W. ciferrii*	NRRL Y-1031^T^	Pod from *Dipteryx odorata*	Dominican Republic	NG_057172	NR_111335
***W. guiyangensis***	**NYNU 2286**^**T**^	**Phylloplane**	**China**	**OP566856**	**OP566860**
	**NYNU 2288**	**Phylloplane**	**China**	**OP581922**	**OP566861**
	**NYNU 22815**	**Phylloplane**	**China**	**OP581928**	**OP566863**
	**NYNU 22852**	**Phylloplane**	**China**	**OP581920**	**OP566864**
*W. edaphicus*	CBS 10408^T^	Forest soil	Thailand	KY110120	KY105904
*W. hampshirensis*	CBS 72088^T^	Insect frass	USA	NG_057168	KY105905
*W. kurtzmanii*	CBS 15418^T^	Crater lake water	China	MK573960	NR_173823
*W. lannaensis*	TBRC 15533^T^	Soil	Thailand	OK135750	MT639220
*W. lynferdii*	NRRL Y-7723^T^	Soil	South Africa	NG_057175	NR_111798
*W. menglaensis*	NYNU 1673^T^	Rotting wood	China	NG_228761	NR_185525
*W. mori*	CBS 12678^T^	Gut of larvae of wood-boring insect	China	NG_064343	NR_160438
*W. mucosus*	CBS 6341^T^	Soil	USA	KY110124	NR_154970
*W. myanmarensis*	CBS 9786^T^	Palm sugar	Myanmar	KY108896	NR_165982
*W. nanensis*	TBRC 15534^T^	Soil	Thailand	OK143510	MT613875
*W. ochangensis*	CBS 11843^T^	Soil	South Korea	HM485464	NR_154971
*W. onychis*	CBS 5587^T^	Nail infection of *Homo sapiens*	Netherlands	POU75421	KY105910
*W. orientalis*	KH-D1^T^	Soil	Iran	KF938676	*KF938677*
***W. paramyanmarensis***	**NYNU 22853**^**T**^	**Phylloplane**	**China**	**OP566871**	**OP566873**
*W. patagonicus*	CBS 11398^T^	Tree sap of *Nothofagus dombeyi*	Argentina	NG_057185	NR_137719
***W. phyllophilus***	**NYNU 2285**^**T**^	**Phylloplane**	**China**	**OP566898**	**OP740375**
	**NYNU 2289**	**Phylloplane**	**China**	**OR727354**	**OR727353**
*W. pijperi*	CBS 2887^T^	Buttermilk	South Africa	KY110127	HM156502
*W. psychrolipolyticus*	Y08-202-2^T^	Soil	Japan	LC333101	—
***W. quanzhouensis***	**NYNU 22496**^**T**^	**Rotting wood**	**China**	**OP269844**	**OP269845**
	**NYNU 224139**	**Rotting wood**	**China**	**OP287955**	**OP287958**
	**NYNU 224203**	**Rotting wood**	**China**	**OP287956**	**OP287961**
*W. queroliae*	CBS 10936^T^	Larva of *Anastrepha mucronata*	Brazil	EU580140	EU580140
*W. rabaulensis*	CBS 6797^T^	Excreta of snail	Papua New Guinea	NG_057165	NR_138207
*W. scolytoplatypi*	CBS 12186^T^	Gallery of *Scolytoplatypus shogun*	Japan	NG_057157	KY105915
*W. siamensis*	DMKU RK359^T^	Phylloplane of *Saccharum officinarum*	Thailand	NG_042337	NR_111029
*W. silvicola*	CBS 1705^T^	Gum of *Prunus serotina*	USA	NG_057164	NR_155012
*W. spegazzinii*	CBS 12756^T^	The fungus garden of *Acromyrmex lundii* nest	Argentina	NG_063933	NR_160053
*W. strasburgensis*	CBS 2939^T^	Leather tanned by vegetable means	France	KY110135	KY105920
*W. subpelliculosus*	NRRL Y-1683^T^	Gut of honey bee	USA	EF550340	NR_111336
*W. sydowiorum*	NRRL Y-7130^T^	Insect frass	South Africa	EF550343	NR_138219
*W. sylviae*	PYCC 6345^T^	Cloaca of *Sylvia communis*	Italy	KF240728	—
*W. tratensis*	CBS 12176^T^	Flower of *Sonneratia caseolaris*	Thailand	KY110150	KY105935
*W. xylosica*	CBS 12320^T^	Soil	Thailand	NG_064304	NR_160310
*W. xylosivorus*	NBRC 111553^T^	Decayed wood	Thailand	NG_057186	NR_155013
*Saccharomyces cerevisiae*	CBS 1171^T^	—	China	JQ689017	NR_111007
*Spathaspora allomyrinae*	CBS 13924^T^	—	China	NG_228732	NR_185513

The newly generated sequences are in bold. Type strains indicated by “T”.

### 2.4 Phylogenetic analysis

A total of 116 nucleotide sequences that belonged to 54 taxa were included in the phylogenetic analyses. Except for 20 sequences recognized in this study, the other sequences were obtained from previous studies (Shimizu et al., 2020; Nundaeng et al., 2021; Chang et al., 2022) and GenBank (Table 2). *Spathaspora allomyrinae* CBS 13924^T^ and *Saccharomyces cerevisiae* CBS 1171^T^ were used as the outgroup. The phylogenetic relationships between *Wickerhamomyces* species and related species were determined through the analysis of combined ITS and LSU datasets. Single gene sequence alignments were generated using CLUSTAL_X v.1.83 (Thompson et al., 1997) or MAFFT 7.110 (Katoh and Standley, 2013) before being manually adjusted with BioEdit v. 7.1.3.0 (Hall, 1999). Multiple genes were concatenated using SequenceMatrix v. 1.7.8 (Vaidya et al., 2011). Gblocks 0.91b was used to detect and remove ambiguously aligned regions from each sequence before phylogenetic analysis (Castresana, 2000). The best-fit evolutionary model was estimated by using Modelfinder (Kalyaanamoorthy et al., 2017).

Phylogenetic analyses were performed using both maximum likelihood (ML) and Bayesian (BI) analyses. ML analysis was performed using RAxML v. 8.2.10 (Stamatakis, 2006) with PHY files generated by CLUSTAL_X v.1.83 (Thompson et al., 1997). The best scoring tree was selected among suboptimal trees from each run by comparing likelihood scores under the GTRGAMMA substitution model. The resulting replicates were plotted on the best scoring tree, which was obtained previously. Statistical support values (BS) were obtained using non-parametric bootstrapping with 1,000 replicates. BI analysis was conducted by MrBayes v. 3.2.7a (Ronquist et al., 2012) in the CIPRES Science Gateway version 3.3. GTR + I + G was selected as the best-fit model for the concatenated dataset. Posterior probabilities (PP) were determined by Markov chain Monte Carlo sampling (MCMC) (Rannala and Yang, 1996). Six simultaneous Markov chains were run for 50 million generations, and the trees were sampled every 1000th generation; thus, 50,000 trees were obtained. The first 25% of the saved trees, representing the burn-in phase of the analysis, were discarded. The remaining trees were used for calculating posterior probabilities (PP) in the majority rule consensus tree.

The phylogenetic trees were viewed with FigTree v. 1.4.3 (Andrew, 2016) and processed with Adobe Illustrator CS5. ML bootstrap support (BS) ≥50% and Bayesian posterior probabilities (PP) ≥0.95 are displayed on the edited phylogenetic tree in the first and second positions, respectively.

## 3 Results

### 3.1 Diversity of *Wickerhamomyces* species

During this investigation, we isolated 36 and 17 yeast strains from rotting wood and plant leaf samples, respectively. Each strain represents the morphology in an individual sample (Table 1). All isolates were identified at the species level based on the threshold of >99% sequence identity with the type strain of described species in the D1/D2 domain or ITS region (Kurtzman and Robnett, 1998; Fell et al., 2000; Vu et al., 2016). In total, we identified 14 distinct species belonging to *Wickerhamomyces*. Among the wood samples, eight known species were represented (*W. anomalus*, *W. arborarius*, *W. ciferrii*, *W. edaphicus*, *W. lynferdii*, *W. pijperi*, *W. subpelliculosa*, and *W. xylosica*) as well as one potentially novel (*Wickerhamomyces* sp. 1). *W. anomalus* and *W. pijperi* were the most dominant, occurring in seven to 10 samples collected across the collection locations. *W. arborarius*, *W. ciferrii*, and *W. subpelliculosa* were the most scarce, occurring only in one sample or location (Table 1). The 17 *Wickerhamomyces* strains collected from plant leaf samples were identified as belong to two known species (*W. strasburgensis* and *W. sydowiorum*) and three potentially new species (*Wickerhamomyces* sp. 2, *Wickerhamomyces* sp. 3, and *Wickerhamomyces* sp. 4). *W. sydowiorum* was the most abundant yeast species among the leaf samples, followed by *Wickerhamomyces* sp. 3, which occurred in four to seven samples (Table 1).

### 3.2 Phylogeny of new yeast species

**Figure 1:**
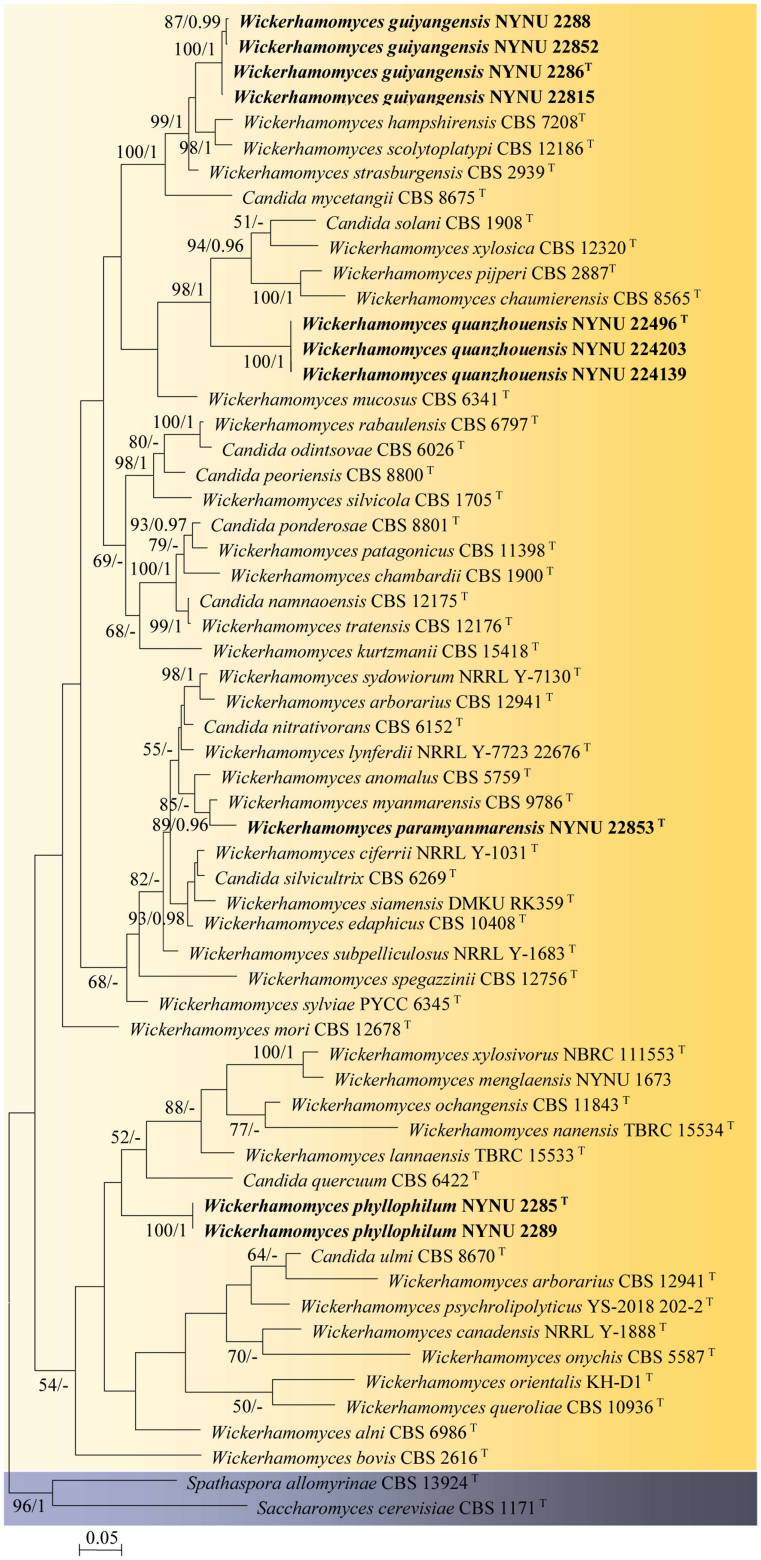
Maximum likelihood phylogram of *Wickerhamomyces* based on the combined ITS and LSU sequence data. *Spathaspora allomyrinae* CBS 13924^T^ and *Saccharomyces cerevisiae* CBS 1171^T^ were used as outgroups. Branches are labeled with BS >50% and PP >0.95, respectively. Strains obtained in the present study are shown in bold.

Among the 53 yeast strains isolated, 10 strains represented 4 new species in the genus *Wickerhamomyces*. To reveal the phylogenetic position of the new species, phylogenetic analyses were performed with a combination of ITS and LSU datasets. A total of 1,276 characteristics, including gaps (ITS: 1–666 and LSU: 667–1,276), were included in the sequence dataset for the phylogenetic analysis. Of these characteristics, 517 were constant, 156 were parsimony-uninformative, and 603 were parsimony-informative. The topology of the ML and Bayesian trees was consistent, therefore only the ML analysis is displayed with BS (≥50%) and PP (≥0.95) at the nodes (Figure 1). In our phylogenetic tree, 10 newly isolated strains were formed into four separate groups within the *Wickerhamomyces* clade, all of which were well supported (100% BS/1 PP) and distinct from other known species.

Four strains isolated from the phylloplane, NYNU 2286^T^, NYNU 2288, NYNU 22815, and NYNU 22852, formed a well-supported clade that clustered with *W. hampshirensis*, *W. scolytoplatypi*, and *W. strasburgensis* with high statistical support (99% BS/1 PP; Figure 1). The four strains of the NYNU 2286^T^ group possess similar sequences with zero to six nt substitutions in the D1/D2 domain and ITS region, indicating that they belong to the same species. BLASTn searches of the D1/D2 and ITS sequences indicate that the NYNU 2286^T^ group is most closely related to type strain of *W. strasburgensis*, differing by 19 nt (~3.3%) substitutions in the D1D2 domain and by 21–29 nt (~3.4%–4.7%) mismatches in the ITS region, respectively. Therefore, the NYNU 2286^T^ group represents a novel *Wickerhamomyces* species, for which the name *W. guiyangensis* sp. nov. is proposed.

Strain NYUN 22853^T^, isolated from the phylloplane, was found to be closely related to *W. myanmarensis* CBS 9786^T^ with strong statistical support (89% BS/0.96 PP; Figure 1). The two strains differed by 7 nt (~1.2%) substitutions in the D1/D2 domain and 19 nt (~3%) mismatches in the ITS region, respectively. These findings suggest that NYUN 22853^T^ represents a novel species in the genus *Wickerhamomyces*, for which the name *W. paramyanmarensis* sp. nov. is proposed.

Three isolates collected from rotting wood, NYNU 22496^T^, NYNU 224139, and NYNU 224203, formed a separate branch, clustering with *C. solani*, *W. xylosica*, *W. pijperi*, and *W. chaumierensis* with good statistical support (98% BS/1 PP; Figure 1). Three isolates of the NYNU 22496^T^ group shared 100% nucleotide identity based on D1/D2 and ITS sequences, indicating that they are conspecific. This group differed from these four known species by 44–53 nt (~7.7–9.3%) substitutions in the D1/D2 domain and more than 26–74 nt (~7.9–11.7%) mismatches in the ITS region. Hence, the NYNU 22496^T^ group represents a novel *Wickerhamomyces* species, for which the name *W. quanzhouensis* sp. nov. is proposed.

Two strains isolated from the phylloplane, NYNU 2285^T^ and NYNU 2289, were located at a basal branch related to *C. quercuum* CBS 6422^T^ but without support (Figure 1). The two strains of the NYNU 2285^T^ group had identical sequences in both the D1/D2 domain and ITS region. This group differed from the closest relative *C. quercuum* CBS 6422^T^ by 20 nt (~3.4%) substitutions in the D1/D2 domain and 18 nt (~5.9%) mismatches in the ITS region, respectively, suggesting that the NYNU 2285^T^ group represents a new species in *Wickerhamomyces*, for which the name *W. phyllophilus* sp. nov. is proposed.

### 3.3 Taxonomy of new yeast species

**Figure 2:**
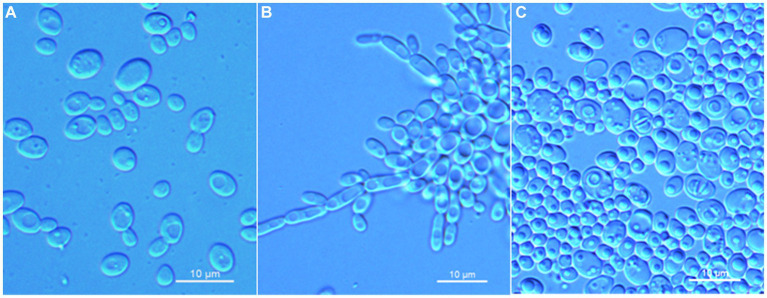
Morphological characteristics of *Wickerhamomyces guiyangensis* sp. nov. (GDMCC 2.320^T^, holotype). **(A)** Budding yeast cells, 3 days, YM broth, 25°C. **(B)** Pseudohyphae, 7 days, CM agar, 25°C. **(C)** Ascospores, 7 days, 5% ME agar, 25°C. Scale bars = 10 μm.

*Wickerhamomyces guiyangensis* C. Y. Chai and F. L. Hui, sp. nov., Figure 2.

MycoBank: MB 850882.

Etymology: the specific epithet “*guiyangensis*” refers to the geographic origin of the type strain, Guiyang city, Guizhou.

Typus: China, Guizhou Province, Guiyang City, Guiyang Botanical Garden, in the phylloplane from undetermined leaf, August 2022, L. Zhang and F. L. Hui, NYUN 2286 (holotype GDMCC 2.320^T^ preserved as a metabolically inactive state, culture ex-type PYCC 9922).

Description: On YM agar, after 7 days at 25°C, colonies are white to cream, raised and butyrous with a smooth surface and an entire margin. In YM broth, after 3 days at 25°C, cells are ovoid or ellipsoidal (3.1–5.9 × 3.5–7.3 μm), occur singly or budding pairs. Budding is multilateral. After 1 month at 25°C, a ring and sediment are present. In Dalmau plate culture on corn meal agar, pseudohyphae are formed, but not true hyphae. Ascospores are observed on 5% ME agar after 7 days of culture at 25°C. D-Glucose and sucrose are fermented, but not D-galactose, maltose, lactose, raffinose, trehalose, or D-xylose. Glucose, inulin, sucrose, raffinose, galactose, trehalose, maltose, melezitose, methyl-α-D-glucoside, cellobiose, salicin, L-rhamnose, D-xylose, L-arabinose, D-arabinose, 5-keto-D-gluconate, glycerol, D-mannitol, D-glucitol, DL-lactate, succinate, citrate, D-gluconate, and D-glucono-1,5-lactone are assimilated as sole carbon sources. Melibiose, lactose, L-sorbose, D-ribose, methanol, ethanol, erythritol, ribitol, galactitol, myo-inositol, D-glucosamine, 2-keto-D-gluconate, and D-glucuronate are not assimilated. Ethylamine and L-lysine are assimilated as sole nitrogen sources. Nitrite, nitrate, and cadaverine are not assimilated. Maximum growth temperature is 35°C. Growth in vitamin-free medium is positive. Growth with 0.01% cylcloheximide, 10% NaCl/5% glucose, and 1% acetic acid is negative. Starch-like compounds are not produced. Diazonium blue B color and urease reaction are negative.

Additional strain examined: China, Guizhou Province, Guiyang City, Guiyang Botanical Garden, in the phylloplane from undetermined leaf, August 2022, L. Zhang and F. L. Hui, NYUN 2288, NYUN 22815 and NYUN 22852.

GenBank accession numbers: holotype GDMCC 2.320^T^ (ITS: OP566860, D1/D2: OP566856); additional strains NYUN 2288 (ITS: OP566861, D1/D2: OP581922), NYUN 22815 (ITS: OP566863, D1/D2: OP581928), and NYUN 22852 (ITS: OP566864, D1/D2: OP581920).

Note: Physiologically, *W. guiyangensis* sp. nov. differed from its close relative *W. strasburgensis* (Kurtzman, 2011b) by its ability to assimilate inulin, D-arabinose, and 5-keto-D-gluconate and its inability to grow in 10% NaCl/5% glucose. Moreover, *W. strasburgensis* weakly ferments galactose and raffinose, while the new species does not.

**Figure 3:**
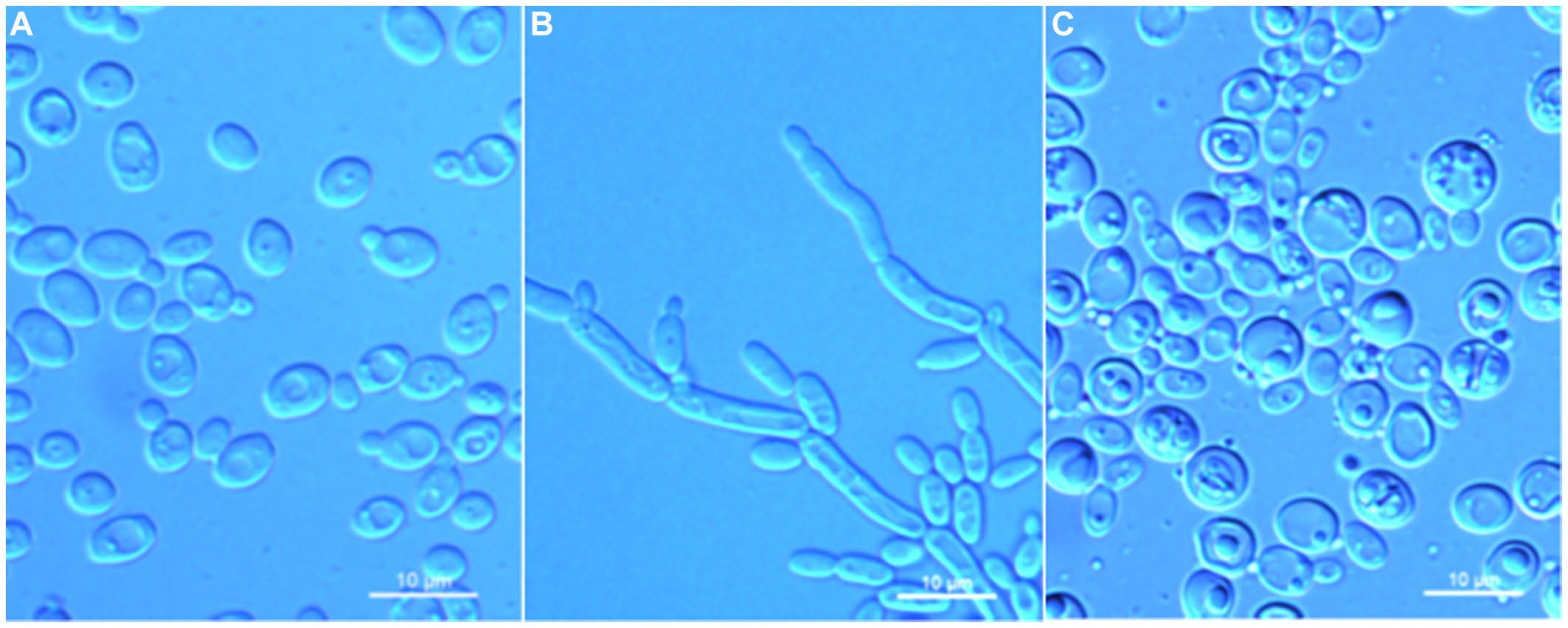
Morphological characteristics of *Wickerhamomyces paramyanmarensis* sp. nov. (GDMCC 2.304^T^, holotype). **(A)** Budding yeast cells, 3 days, YM broth, 25°C. **(B)** Pseudohyphae, 7 days, CM agar, 25°C. **(C)** Ascospores, 7 days, 5% ME agar, 25°C. Scale bars = 10 μm.

*Wickerhamomyces paramyanmarensis* C. Y. Chai and F. L. Hui, sp. nov., Figure 3.

MycoBank: MB 850883.

Etymology: the specific epithet “*paramyanmarensis*” refers to its phylogenetic closeness to *W. myanmarensis*.

Typus: China, Guizhou Province, Guiyang City, Guiyang Botanical Garden, in the phylloplane from undetermined leaf, August 2022, L. Zhang and F. L. Hui, NYNU 22853 (holotype GDMCC 2.304^T^ preserved as a metabolically inactive state, culture ex-type PYCC 9924).

Description: On YM agar, after 7 days at 25°C, colonies are white to cream, raised and butyrous with a smooth surface and an entire margin. In YM broth, after 3 days at 25°C, cells are ovoid or ellipsoidal (3.5–4.9 × 4.6–7.2 μm), occur singly or budding pairs. Budding is multilateral. After 1 month at 25°C, a ring and sediment are present. In Dalmau plate culture on corn meal agar, pseudohyphae are formed but not true hyphae. Ascospores are observed on 5% ME agar after 7 days of culture at 25°C. D-Glucose and sucrose are fermented but not D-galactose, maltose, lactose, raffinose, trehalose, or D-xylose. Glucose, inulin, sucrose, raffinose, melibiose, galactose, lactose, trehalose, maltose, melezitose, methyl-α-D-glucoside, cellobiose, L-rhamnose, D-xylose, L-arabinose, D-ribose, glycerol, erythritol, D-mannitol, D-glucitol, DL-lactate, D-gluconate, 2-keto-D-gluconate, and D-glucuronate are assimilated as sole carbon sources. Salicin, L-sorbose, D-arabinose, 5-keto-D-gluconate, methanol, ethanol, ribitol, galactitol, myo-inositol, succinate, citrate, D-glucosamine, and glucono-1,5-lactone are not assimilated. Nitrite, nitrate, ethylamine, L-lysine, and cadaverine are not assimilated. Maximum growth temperature is 35°C. Growth in vitamin-free medium is negative. Growth with 0.01% cylcloheximide, 10% NaCl/5% glucose, and 1% acetic acid is negative. Starch-like compounds are not produced. Diazonium blue B color and urease reaction are negative.

GenBank accession numbers: holotype GDMCC 2.304^T^ (ITS: OP566873, D1/D2: OP566871).

Note: Physiologically, *W. paramyanmarensis* sp. nov. differed from its close relative *W. myanmarensis* (Kurtzman, 2011a) in the ability to assimilate inulin, melibiose, lactose, and L-rhamnose and inability to assimilate salicin, D-arabinose, ethanol, ribitol, succinate, and citrate and growth at 37°C. Moreover, *W. myanmarensis* latently ferments galactose, maltose, and raffinose, while the new species does not.

**Figure 4:**
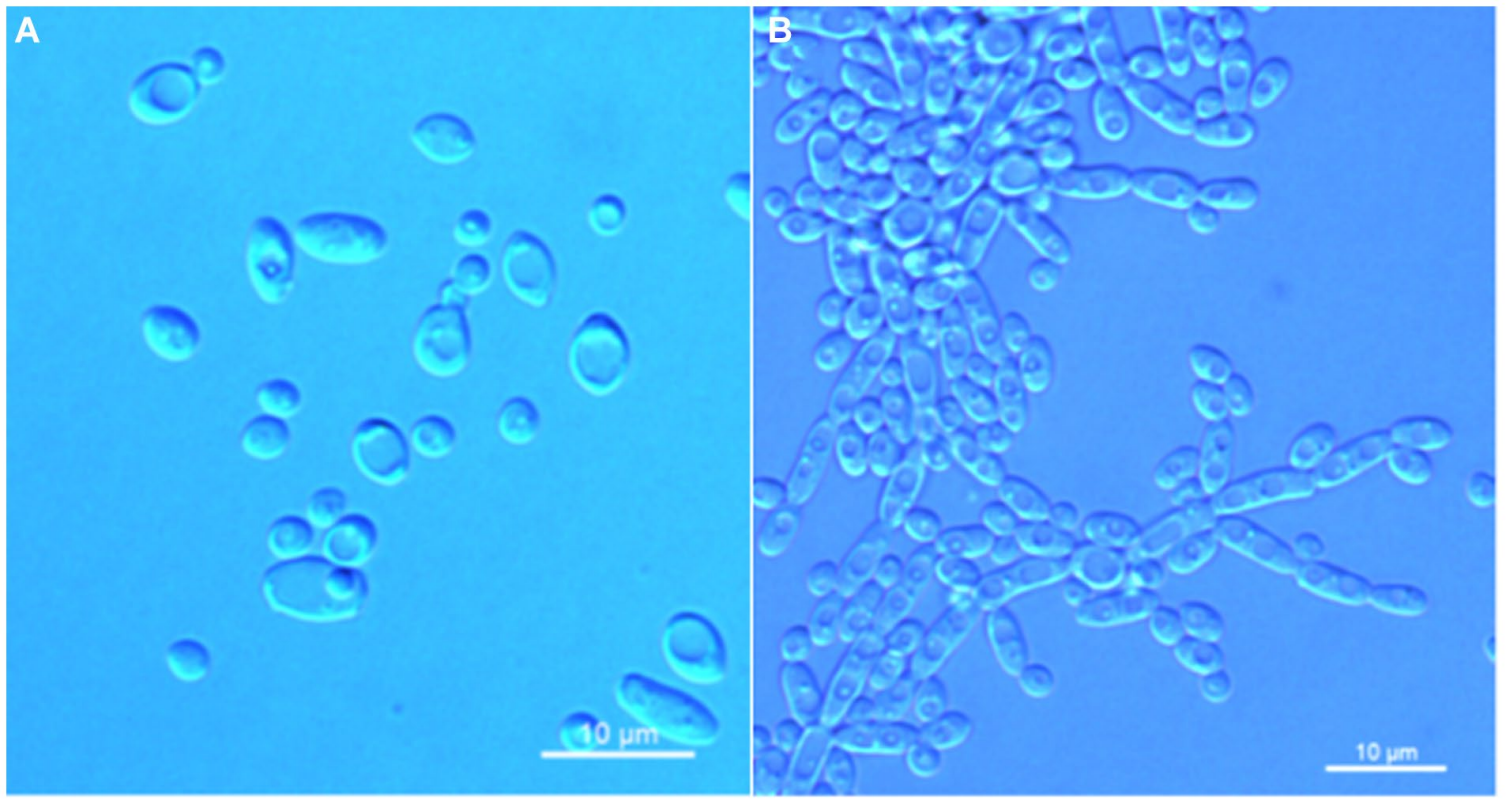
Morphological characteristics of *Wickerhamomyces quanzhouensis* sp. nov. (GDMCC 2.308^T^, holotype). **(A)** Budding yeast cells, 3 days, YM broth, 25°C. **(B)** Pseudohyphae, 7 days, CM agar, 25°C. Scale bars = 10 μm.

*Wickerhamomyces quanzhouensis* C. Y. Chai and F. L. Hui, sp. nov., Figure 4.

MycoBank: MB 850884.

Etymology: the specific epithet “*quanzhouensis*” refers to the geographic origin of the type strain, Quanzhou city, Fujian.

Typus: China, Fujian Province, Quanzhou City, Qingyuan Mountain, in rotting wood, April 2022, W. T. Hu and S. B. Chu, NYNU 22496 (holotype GDMCC 2.308^T^ preserved as a metabolically inactive state, culture ex-type PYCC 9923).

Description: On YM agar, after 7 days at 25°C, colonies are white to cream color, raised and butyrous with a smooth surface and an entire margin. In YM broth, after 3 days at 25°C, cells are ovoid to ellipsoid (2.0–3.7 × 2.6–7.5 μm), occur singly or budding pairs. Budding is multilateral. After 1 month at 25°C, a ring and sediment are present. In Dalmau plate culture on corn meal agar, pseudohyphae are formed but not true hyphae. Ascospores were not obtained for individual strains and strain pairs on YM agar, CM agar, 5% ME agar, and V8 agar at 20°C and 25°C for 2 months. D-Glucose is fermented but not D-galactose, sucrose, maltose, lactose, raffinose, trehalose, or D-xylose. Glucose, inulin, sucrose, raffinose, trehalose, maltose, cellobiose, salicin, D-xylose, D-arabinose, ethanol, glycerol, ribitol, galactitol, D-mannitol, D-glucitol, DL-lactate, succinate, citrate, and glucono-1,5-lactone are assimilated as sole carbon sources. Melibiose, galactose, lactose, melezitose, methyl-α-D-glucoside, L-sorbose, L-rhamnose, L-arabinose, D-ribose, methanol, erythritol, myo-inositol, D-gluconate, D-glucosamine, 2-keto-D-gluconate, and D-glucuronate are not assimilated. Ethylamine and L-lysine are assimilated as sole nitrogen sources. Nitrite, nitrate, and cadaverine are not assimilated. Maximum growth temperature is 30°C. Growth in vitamin-free medium is positive. Growth with 0.01% cylcloheximide, 10% NaCl/5% glucose, and 1% acetic acid is negative. Starch-like compounds are not produced. Diazonium blue B color and urease reaction are negative.

Additional strain examined: China, Fujian Province, Quanzhou City, Qingyuan Mountain, in rotting wood, April 2022, W. T. Hu and S. B. Chu, NYUN 224139 and NYUN 224203.

GenBank accession numbers: holotype GDMCC 2.308^T^ (ITS: OP269845, D1/D2: OP269844); additional strains NYUN 224139 (ITS: OP287958, D1/D2: OP287955) and NYUN 224203 (ITS: OP287961, D1/D2: OP287956).

Note: Physiologically, *W. quanzhouensis* sp. nov. differed from its close relative *W. xylosica* (Limtong et al., 2012) in the ability to assimilate inulin, raffinose, D-arabinose, ribitol, galactitol, D-glucitol, and citrate and inability to assimilate melezitose, L-sorbose, and 2-keto-D-gluconate.

**Figure 5:**
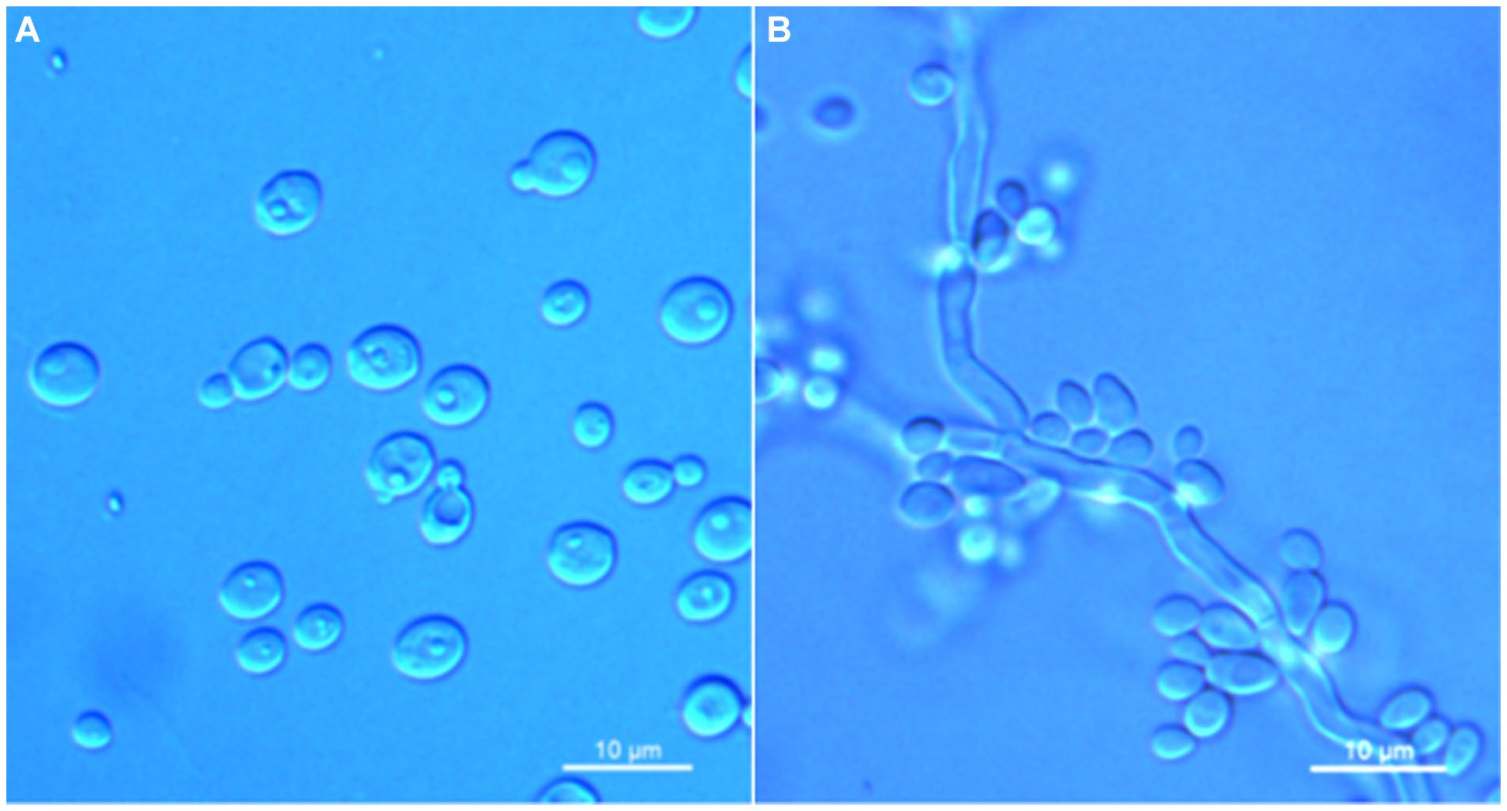
Morphological characteristics of *Wickerhamomyces phyllophilus* sp. nov. (GDMCC 2.301^T^, holotype). **(A)** Budding yeast cells, 3 days, YM broth, 25°C. **(B)** Pseudohyphae, 7 days, CM agar, 25°C. Scale bars = 10 μm.

*Wickerhamomyces phyllophilus* C. Y. Chai and F. L. Hui, sp. nov., Figure 5.

MycoBank: MB 850885.

Etymology: the specific epithet “*phyllophilus*” refers to leaves, the substrate origin of the type strain.

Typus: China, Guizhou Province, Guiyang City, Guiyang Botanical Garden, in the phylloplane from leaf of Celtis sp., August 2022, L. Zhang and F. L. Hui, NYNU 2285 (holotype GDMCC 2.301^T^ preserved as a metabolically inactive state, culture ex-type PYCC 9921).

Description: On YM agar, after 7 days at 25°C, colonies are white to cream color, raised, and butyrous with a smooth surface and an entire margin. In YM broth, after 3 days at 25°C, cells are ovoid to ellipsoid (3.4–5.7 × 3.6–6.7 μm), occur singly or budding pairs. Budding is multilateral. After 1 month at 25°C, a ring and sediment are present. In Dalmau plate culture on corn meal agar, pseudohyphae are formed but not true hyphae. Ascospores were not obtained for individual strains and strain pairs on YM agar, CM agar, 5% ME agar, and V8 agar at 20°C and 25°C for 2 months. D-Glucose is fermented but not D-galactose, sucrose, maltose, lactose, raffinose, trehalose, or D-xylose. Glucose, inulin, sucrose, galactose, trehalose, maltose, melezitose, methyl-α-D-glucoside, cellobiose, D-xylose, glycerol, D-mannitol, D-glucitol, DL-lactate, and D-gluconate (weak) are assimilated as sole carbon sources. Raffinose, melibiose, lactose, salicin, L-sorbose, L-rhamnose, L-arabinose, D-arabinose, 5-keto-D-gluconate, D-ribose, methanol, ethanol, erythritol, ribitol, galactitol, myo-inositol, succinate, citrate, D-glucosamine, 2-keto-D-gluconate, D-glucuronate, and glucono-1,5-lactone are not assimilated. Nitrite, nitrate, ethylamine, L-lysine, and cadaverine are not assimilated. Maximum growth temperature is 35°C. Growth in vitamin-free medium is negative. Growth with 0.01% cylcloheximide, 10% NaCl/5% glucose, and 1% acetic acid is negative. Starch-like compounds are not produced. Diazonium blue B color and urease reaction are negative.

Additional strain examined: China, Guizhou Province, Guiyang City, Guiyang Botanical Garden, in the phylloplane from leaf of *Celtis* sp., August 2022, L. Zhang and F. L. Hui, NYUN 2289.

GenBank accession numbers: holotype GDMCC 2.301^T^ (ITS: OP740375, D1/D2: OP566898); additional strains NYUN 2289 (ITS: OR727354, D1/D2: OR727353).

Note: Physiologically, *W. phyllophilus* sp. nov. differed from its close relative *C. quercuum* (Lachance et al., 2011) in the ability to assimilate inulin, galactose, and trehalose and inability to assimilate salicin, ethanol, succinate, and citrate.

## 4 Discussion

Before advances in gene sequencing, the identification of *Wickerhamomyces* species was primarily based on phenotypic characteristics. However, due to the presence of many shared polymorphic characteristics and similar appearances across different species, this method is often inaccurate (Nundaeng et al., 2021). Now, ribosomal DNA gene sequence analysis has become the dominant form of identification as it has proven to be very effective (Vu et al., 2016). Molecular taxonomic studies have deeply improved our understanding of the phylogenetic relationships, systematics, and ecology of yeasts. As a result, a combination of phenotypic and phylogenetic data is used to concretely identify the *Wickerhamomyces* species (Kurtzman and Robnett, 1998; Nundaeng et al., 2021). In this study, we introduced four new *Wickerhamomyces* species, consisting of *W. guiyangensis* sp. nov., *W. paramyanmarensis* sp. nov., *W. quanzhouensis* sp. nov., and *W. phyllophilus* sp. nov., and describe them in terms of both phenotype and phylogeny. These new species formed four well-separated clades in the resultant phylogram, which indicated the distinct phylogenetic positions of each species within the genus *Wickerhamomyces*. Pairwise sequence comparisons of the D1/D2 domain and the ITS region between these four species and closely related species showed that they had lower similarity values than the common threshold for species demarcation in ascomycetous yeasts (Fell et al., 2000; Kurtzman et al., 2008; Vu et al., 2016). However, they were found to be highly similar in cell shape, colony morphology, and color, but they differed from the closest related species in terms of their physiological and biochemical characteristics. Therefore, a combination of phenotypic characteristics and molecular phylogenetic analyses conducted in our study confirmed the existence of these new species in China.

Before our study, only four Chinese species, *W. anomalus*, *W. kurtzmanii*, *W. menglaensis*, and *W. mori*, were known from China (Hui et al., 2013; You et al., 2016; Chai et al., 2019; Zhou et al., 2019). This study provides 14 additional species, including 1 previously known species (*W. anomalus*), 9 new recorded species (*W. arborarius*, *W. ciferrii*, *W. edaphicus*, *W. lynferdii*, *W. pijperi*, *W. subpelliculosa*, *W. xylosica*, *W. strasburgensis*, and *W. sydowiorum*), and 4 novel species (*W. guiyangensis* sp. nov., *W. paramyanmarensis* sp. nov., *W. quanzhouensis* sp. nov., and *W. phyllophilus* sp. nov.), increasing the number of *Wickerhamomyces* species from 4 to 17. In China, there are likely species that need to be identified, such as GenBank accession JQ901898. These studies suggest that there are likely even more *Wickerhamomyces* species waiting to be discovered. So far, almost no *Wickerhamomyces* species have been reported from eastern China, which also have abundant forest resources, so it is necessary to investigate yeast resources in these regions in the future studies.

Members of the *Wickerhamomyces* clade are widely distributed and are found in different habitats, as shown in Table 2. They can be successfully isolated from soil (Limtong et al., 2012; Shimizu et al., 2020), phylloplane (Kaewwichian et al., 2013; Into et al., 2020), tree exudates (Kurtzman, 2011b), flowers (Nakase et al., 2012), rotting wood (Kobayashi et al., 2017; Chai et al., 2019), insect (Hui et al., 2013), insect frass (Nakase et al., 2012), birds (Kurtzman, 2011b), patients (Kurtzman, 2011b; Arastehfar et al., 2019), fermented food (Ravasio et al., 2018; Yan et al., 2019; Li et al., 2021), brined vegetables (Kurtzman, 2011b), and crater lake water (Zhou et al., 2019), but most known species are found mostly in association with plant materials. However, only a few species, such as *W. edaphicus*, *W. siamensis*, *W. menglaensis*, and *W. xylosivorus*, have been isolated from rotting wood and plant leaf samples, respectively. In this study, we isolated nine species (*W. anomalus*, *W. arborarius*, *W. ciferrii*, *W. edaphicus*, *W. lynferdii*, *W. pijperi*, *W. quanzhouensis* sp. nov., *W. subpelliculosa*, and *W. xylosica*) from rotting wood and an additional five species (*W. guiyangensis* sp. nov., *W. paramyanmarensis* sp. nov., *W. phyllophilus* sp. nov., *W. strasburgensis*, and *W. sydowiorum*) from plant leaves. Previous studies showed that phylloplane yeast strains of *W. anomalus* have a variety of biological functions, such as resistance to plant pathogenic fungi and biosynthesis of the plant growth promoter indole-3-acitic acid (IAA) (Kumla et al., 2018; Czarnecka et al., 2019; Limtong et al., 2020). In this study, we also isolated 17 strains of *Wickerhamomyces* from the phylloplane, which may own similar ecological functions as *W. anomalus*. In addition, D-xylose assimilation was observed for *W. quanzhouensis* sp. nov., which was similar to those found for *W. menglaensis* and *W. xylosivorus* (Kobayashi et al., 2017; Chai et al., 2019). D-xylose assimilation may be an important physiological trait for these yeasts in the colonization of rotting wood. These findings expanded our knowledge of where *Wickerhamomyces* species can survive and demonstrates the complicated ecological function of this genus. Future research will focus on *Wickerhamomyces* diversity from a broader range of substrates and environments. Ultimately, these findings will help researchers gain a better understanding of the diversity, distribution, and ecology of *Wickerhamomyces*.

## 5 Conclusion

In this study, the species diversity of the genus *Wickerhamomyces* in China was studied. A total of 14 species were obtained and circumscribed as four novel species, namely, *W. guiyangensis* sp. nov., *W. paramyanmarensis* sp. nov., *W. quanzhouensis* sp. nov., and *W. phyllophilus* sp. nov., nine newly recorded species *W. arborarius*, *W. ciferrii*, *W. edaphicus*, *W. lynferdii*, *W. pijperi*, *W. subpelliculosa*, *W. xylosica*, *W. strasburgensis*, and *W. sydowiorum* in China, and a known species *W. anomalus*. All the studied species were identified by morphological characteristics and phylogenetic analysis of combined ITS and LSU sequences. Four new species are described based on their asexual and/or sexual states, and their differences with the close relatives were compared and discussed.

## Data availability statement

The datasets presented in this study can be found in online repositories. The names of the repository/repositories and accession number(s) can be found in the article/supplementary material.

## Author contributions

C-YC: Investigation, Methodology, Writing – original draft. TK: Investigation, Methodology, Resources, Software, Writing – original draft. Q-HN: Funding acquisition, Resources, Software, Validation, Writing – review & editing. F-LH: Resources, Writing – review & editing.
